# Acetylcholinesterase as a Multifunctional Target in Amyloid-Driven Neurodegeneration: From Dual-Site Inhibitors to Anti-Agregation Strategies

**DOI:** 10.3390/ijms26178726

**Published:** 2025-09-07

**Authors:** Weronika Grabowska, Michal Bijak, Rafał Szelenberger, Leslaw Gorniak, Marcin Podogrocki, Piotr Harmata, Natalia Cichon

**Affiliations:** 1 Biohazard Prevention Centre, Faculty of Biology and Environmental Protection, University of Lodz, Pomorska 141/143, 90-236 Lodz, Poland; weronika.grabowska@biol.uni.lodz.pl (W.G.); michal.bijak@biol.uni.lodz.pl (M.B.); rafal.szelenberger@biol.uni.lodz.pl (R.S.); leslaw.gorniak@biol.uni.lodz.pl (L.G.); marcin.podogrocki@biol.uni.lodz.pl (M.P.); 2 Faculty of Advanced Technologies and Chemistry, Military University of Technology, 2 gen. S. Kaliskiego St., 00-908 Warsaw, Poland; piotr.harmata@wat.edu.pl

**Keywords:** acetylcholinesterase inhibitors, multi-target-directed ligands, amyloid aggregation, dual-site inhibitors, neurodegeneration

## Abstract

Acetylcholinesterase (AChE) has emerged not only as a cholinergic enzyme but also as a modulator of β-amyloid (Aβ) aggregation via its peripheral anionic site (PAS), making it a dual-purpose target in Alzheimer’s disease. While classical AChE inhibitors provide symptomatic relief, they lack efficacy against the amyloidogenic cascade. This review highlights recent advances in multifunctional AChE pharmacophores that inhibit enzymatic activity while simultaneously interfering with Aβ aggregation, oxidative stress, metal dyshomeostasis, and neuroinflammation. Particular emphasis is placed on dual-site inhibitors targeting both catalytic and peripheral domains, multi-target-directed ligands (MTDLs) acting on multiple neurodegenerative pathways, and metal-chelating hybrids that address redox-active metal ions promoting Aβ fibrillization. We also discuss enabling technologies such as AI-assisted drug design, high-resolution structural tools, and human induced pluripotent stem cell (iPSC)-derived neuronal models that support physiologically relevant validation. These insights reflect a paradigm shift towards disease-modifying therapies that bridge molecular pharmacology and pathophysiological relevance.

## 1. Introduction

Acetylcholinesterase (AChE, E.C. 3.1.1.7) is an enzyme involved in cholinergic neurotransmission, where it plays a key role in the breakdown of the neurotransmitter acetylcholine (ACh) into choline and acetate [1]. This rapid breakdown of ACh terminates synaptic signalling, ensuring tight regulation of cholinergic neurotransmission. In the central nervous system (CNS), this mechanism is especially important for maintaining cognitive processes such as learning, attention, and memory [2]. Disruption of this tightly regulated system, as seen in Alzheimer’s disease (AD) and other neurodegenerative disorders, leads to impaired cholinergic signalling, cognitive decline, and progressive neuronal dysfunction [3].

Due to its central role in cholinergic signalling, AChE has long been a target for therapeutic intervention, particularly in the treatment of AD [4,5]. Several AChE inhibitors, including donepezil, galantamine, and rivastigmine, have been approved for clinical use and offer modest symptomatic relief [6]. These agents work by increasing the concentration of ACh at synaptic junctions, thereby temporarily enhancing cholinergic transmission. However, their therapeutic impact is limited. Many of these drugs suffer from poor selectivity, limited blood–brain barrier (BBB) permeability, and dose-dependent side effects such as gastrointestinal distress, hepatotoxicity, and bradycardia [7,8]. More importantly, they do not modify the underlying neurodegenerative processes and therefore fail to halt or reverse disease progression. In recent years, there has been a significant shift in the approach to AChE-targeted therapy. Rather than focusing solely on inhibiting enzymatic activity, researchers are now designing compounds that also address other pathological mechanisms involved in neurodegeneration [9,10]. AD, for example, involves a complex network of processes including oxidative stress, β-amyloid (Aβ) aggregation, tau hyperphosphorylation, mitochondrial dysfunction, and neuroinflammation [9]. This has led to the development of multi-target-directed ligands (MTDLs), which are rationally designed molecules that can interact with multiple biological targets simultaneously [11,12,13]. In this broader therapeutic context, AChE is increasingly being considered not only as a target for symptom relief but also as a strategic scaffold for multifunctional ligands.

Structural and computational advances have made this shift possible. High-resolution crystallographic data have revealed that AChE contains at least two major binding domains: a catalytic active site (CAS) and a peripheral anionic site (PAS). While the CAS is responsible for hydrolysing ACh, the PAS is involved in modulating interactions with other substrates, including the aggregation of Aβ peptides [14]. This dual-binding architecture has opened new avenues for drug design, enabling the development of inhibitors that can bind both sites simultaneously [15]. These compounds may not only enhance cholinergic signalling but also interfere with key steps in amyloid plaque formation.

In parallel, progress in computational pharmacophore modelling, virtual screening, and artificial intelligence has accelerated the discovery of novel AChE inhibitors [16,17]. Several novel ligands have emerged from computational and hybrid design approaches, showing diverse potencies against AChE. Among the most active are derivatives of already approved AChE inhibitors, as well as other novel derivatives [18,19], that show low nanomolar or sub-nanomolar inhibition in vitro [20,21] and, in some cases, demonstrate multifunctional effects such as interference with Aβ aggregation or modulation of additional enzymatic targets [22,23]. However, very few multifunctional AChE inhibitors have progressed to clinical trials [9,24,25], highlighting the ongoing challenge of translating preclinical efficacy into safe and effective therapies for humans.

Natural products and their derivatives have also received renewed interest, offering structurally diverse scaffolds with inherent biological activity and the potential for multifunctional action [26,27].

Therefore, the aim of this review is to critically examine recent advances in acetylcholinesterase pharmacophore design, with particular emphasis on multifunctional ligands capable of modulating both cholinergic and non-cholinergic pathological pathways in neurological diseases. By analysing structural features, dual-site targeting strategies, multi-target-directed ligand frameworks, and emerging modalities such as metal chelation, photoactivation, and prodrug design, this work seeks to highlight key innovations and remaining challenges in the development of next-generation AChE inhibitors. Furthermore, the review aims to provide a conceptual framework for the rational design of ligands with enhanced efficacy, selectivity, and CNS bioavailability.

## 2. Structural and Functional Characteristics of Acetylcholinesterase

**Figure 1:**
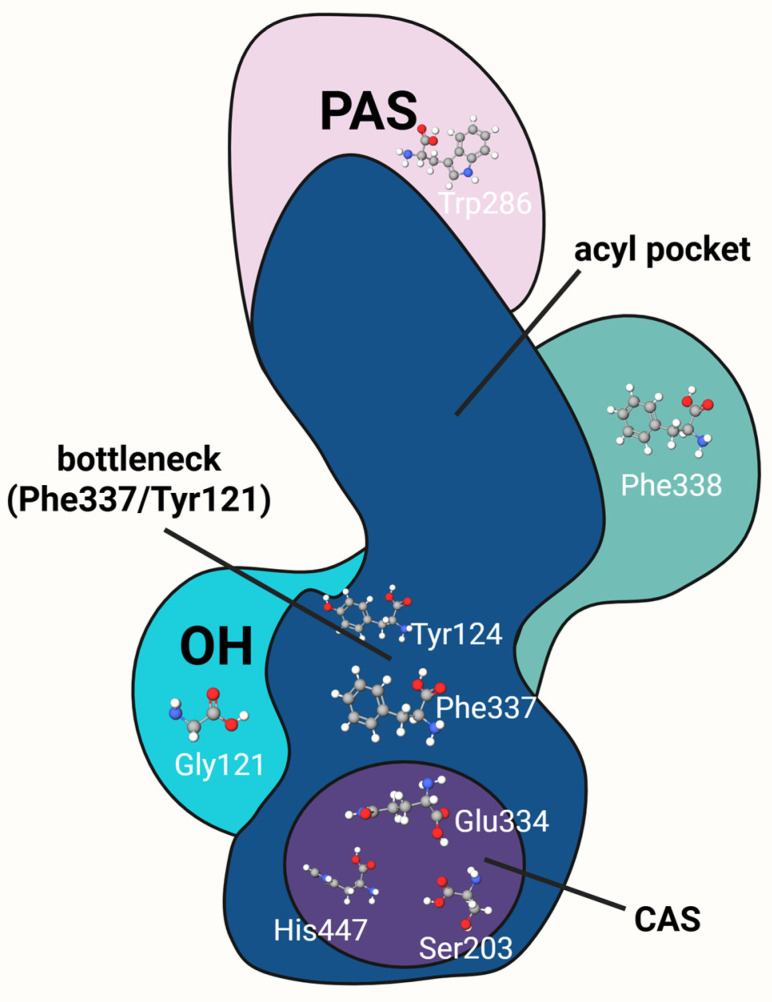
Structural domains of AChE along the catalytic gorge. Schematic cross-section of AChE highlighting key functional regions: CAS comprising Ser203, Glu334, and His447; the PAS with Trp286; the acyl pocket (Phe338); the oxyanion hole (OH) with Gly121; and the bottleneck formed by Phe337 and Tyr121. The spatial arrangement of these domains enables substrate recognition, stabilization, and hydrolysis. Created in BioRender. Bijak, M. (2025) https://BioRender.com/zsqb6gn.

The therapeutic significance of AChE arises not only from its pivotal role in cholinergic neurotransmission but also from its unique structural features that underlie exceptional catalytic efficiency. A thorough understanding of these properties is essential for the rational design of inhibitors with high selectivity, CNS engagement, and functional specificity [28,29]. To better illustrate the spatial organization of AChE and the role of its active site domains in substrate recognition and catalysis, Figure 1 and Table 1 present a schematic cross-section of the enzyme’s catalytic gorge.

AChE is a globular enzyme with a highly conserved tertiary structure across species. The enzyme’s active site is located at the base of a ~20 Å deep gorge, which serves as the ligand-binding site of the enzyme [29]. Within this gorge, the enzyme catalyses the hydrolysis of the ligand or interacts with it in a manner that leads to enzyme inhibition. AChE activity is inhibited when a ligand binds to specific regions within the gorge—either the catalytic active site or peripheral domains—thereby disrupting or completely blocking the catalytic cycle. Inhibitors may mimic the natural substrate, forming stable interactions with amino acid residues in the catalytic centre, or bind to the PAS, hindering the proper positioning and translocation of ACh toward the catalytic active site. Depending on the nature of these interactions, various stages of the enzymatic mechanism may be inhibited, ultimately leading to ACh accumulation in the synaptic cleft and disruption of cholinergic neurotransmission.

The gorge extends from the enzyme surface to the catalytic site and comprises several key functional domains: PAS, CAS, OH, and the acyl pocket, which binds acyl groups and contributes to substrate selectivity [30]. The gorge is lined with multiple aromatic amino acid residues, among which tryptophan W86 and phenylalanine F337 (numbering according to human AChE) play major roles in stabilizing the ACh molecule upon binding [35]. This stabilization is further supported by cation–π interactions between the quaternary ammonium group of ACh and the aromatic residues. A structurally distinctive feature of the gorge is the so-called “bottleneck” formed by F337 and Y124. Despite its narrow width, this region demonstrates conformational flexibility, allowing the enzyme to adapt its shape for effective substrate binding.

The PAS, located near the entrance of the gorge, is a key structural element in the initial stages of substrate recognition. It includes residues such as tyrosines Y72, Y124, and Y341, asparagine B74, and tryptophan W286, arranged around the mouth of the gorge [14]. Residues Y124, Y341, and W286 are involved in interactions with positively charged groups of the substrate, including the quaternary amine of ACh [32]. These interactions help guide the substrate deeper into the gorge, directing it toward the CAS. In addition, W286 contributes to binding lipophilic moieties present in substrates, further stabilizing their positioning within the gorge [33].

Kinetic studies indicate that the CAS is composed of two primary sub-sites: the esteratic site and the anionic site [30]. The esteratic site contains the catalytic triad—serine S203, histidine H447, and glutamic acid E334—where serine and histidine directly participate in ester bond hydrolysis through proton transfer, while glutamate stabilizes the transition state. The anionic site binds positively charged groups, including the quaternary amine of ACh or other cations, thereby stabilizing the substrate within the active site [31].

The OH, located near the base of the gorge, is formed by glycine residues G121 and G122, as well as A204. It interacts with the negatively charged oxygen atom (oxyanion) that forms during catalysis by stabilizing the negative charge of the transition state (enzyme–substrate complex) [34]. This stabilization lowers the activation energy of the reaction and enhances the enzyme’s catalytic efficiency.

During catalysis, the carbon–oxygen double bond in the acetyl group of ACh is cleaved, forming a transient covalent complex between the hydroxyl group of serine S203 and the carbon atom of the substrate’s carbonyl group. This results in a non-covalent transition state, in which the oxyanion is stabilized by interactions with the amide groups in the oxyanion hole, reducing the activation energy of the reaction. Subsequently, the bond between the choline and acetyl groups is cleaved, releasing choline and forming an intermediate—acetylated serine (CH_3_CO–AChE). In the final step, the acetyl–serine bond is hydrolysed by a water molecule, leading to the release of acetic acid and regeneration of the active enzyme, ready for another catalytic cycle [36,37]. From a drug development standpoint, this mechanism provides multiple points for therapeutic intervention, whether by blocking substrate access, mimicking transition states, or altering the conformation of key active-site elements.

## 3. Approved Acetylcholinesterase Inhibitors and Their Limitations

**Figure 2:**
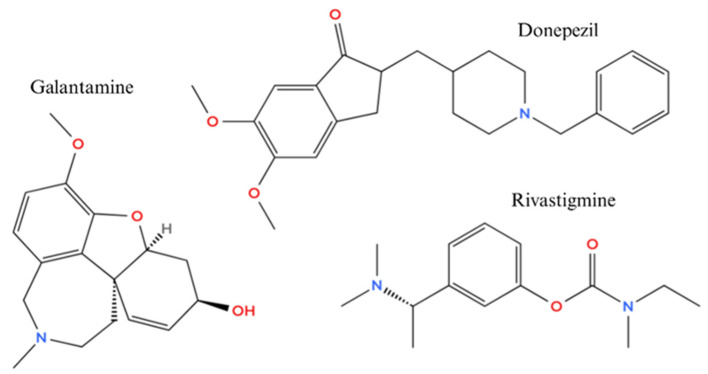
Chemical structures of the AChE: inhibitors galantamine, donepezil, and rivastigmine. Created in BioRender. Bijak, M. (2025) https://BioRender.com/85f6vfp.

Donepezil, galantamine, and rivastigmine are the only acetylcholinesterase inhibitors currently approved by both the FDA and EMA for the treatment of Alzheimer’s disease (Figure 2) [38,39].

These agents have demonstrated the ability to temporarily improve cognitive function by increasing acetylcholine levels in the synaptic cleft, thereby addressing the hallmark cholinergic deficits observed in neurodegenerative conditions [40]. However, despite their clinical utility, these inhibitors are limited by several significant challenges that have spurred ongoing research into more advanced pharmacophores. Their effects are largely symptomatic and do not halt or reverse the progression of underlying pathology (Table 2) [41,42]. Moreover, their lack of selectivity often results in peripheral side effects such as nausea, vomiting, and hepatotoxicity, which constrain dosing and impact patient adherence [43]. Many inhibitors face difficulties crossing the blood–brain barrier efficiently and exhibit pharmacokinetic profiles that complicate chronic administration. Importantly, by focusing solely on acetylcholinesterase activity, these compounds fail to address the multifactorial nature of Alzheimer’s and related neurodegenerative diseases, where amyloid plaque formation, tau pathology, oxidative stress, and neuroinflammation all contribute to disease progression [44]. These limitations have fuelled the transition toward the design of multifunctional pharmacophores that not only inhibit AChE but also target other pathological mechanisms, offering hope for disease-modifying therapies with improved efficacy and safety profiles [45,46]. This evolution reflects a broader paradigm shift in drug discovery, emphasizing the need for integrated approaches capable of tackling the complexity of neurodegeneration.

## 4. New Trends in AChE Pharmacophores

The limitations of approved acetylcholinesterase inhibitors have driven innovation toward new pharmacophore designs that go beyond simple enzyme blockade. Recent advances emphasize the development of multifunctional agents capable of simultaneously modulating several pathological targets involved in neurodegenerative diseases [50,51,52,53]. These emerging compounds aim not only to enhance cholinergic neurotransmission but also to interfere with Aβ aggregation, reduce oxidative stress, and attenuate neuroinflammation, addressing the complex interplay of mechanisms underlying conditions like AD.

**Figure 3:**
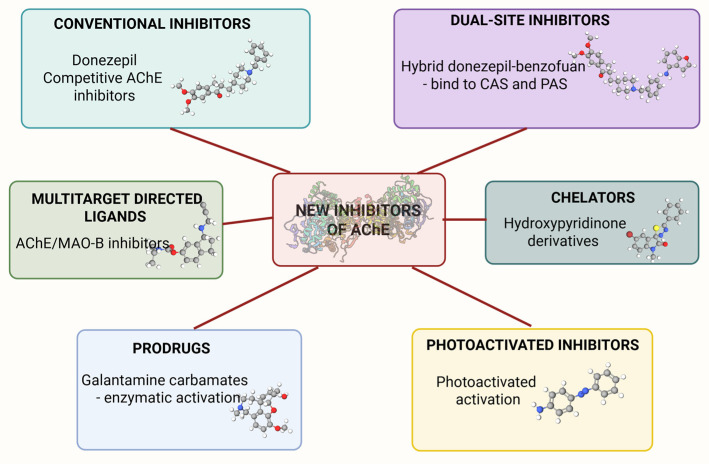
Classification of new AChE inhibitors based on multifunctional design strategies. Overview of novel acetylcholinesterase inhibitors categorized into six classes: (1) conventional inhibitors (e.g., donepezil); (2) dual-site inhibitors targeting both CAS and PAS (e.g., donepezil–benzofuran hybrids); (3) multi-target-directed ligands (MTDLs) acting on AChE and other enzymes like MAO-B; (4) metal-chelating agents (e.g., hydroxypyridinone derivatives); (5) prodrugs (e.g., galantamine carbamates) activated enzymatically in the CNS; and (6) photoactivated inhibitors designed for light-triggered activity modulation. Created in BioRender. Bijak, M. (2025) https://BioRender.com/3ir0qvn.

As research efforts have evolved beyond classical enzyme inhibition, novel AChE pharmacophores are now being classified according to their multifunctional capabilities and mechanisms of action. Figure 3 summarizes the six main categories of emerging AChE inhibitors—including conventional, dual-site, MTDLs, metal-chelating, prodrug, and photoactivated compounds—alongside representative examples and molecular mechanisms.

One of the most prominent trends is MLDs, which involves engineering single molecules capable of interacting with multiple disease-relevant targets. For AChE inhibitors, this often means combining cholinergic enhancement with antioxidant activity, anti-amyloid aggregation potential, or metal chelation [54].

Sub-strategy within this paradigm is dual-site targeting, where ligands simultaneously bind both the CAS and PAS of AChE. Particular emphasis has been placed on compounds that not only inhibit AChE activity but also interfere with Aβ aggregation, thereby improving inhibitory potency while disrupting the enzyme’s pro-amyloid function [55]. This approach is supported by the well-established role of the PAS in accelerating Aβ fibrillogenesis by serving as a nucleation site for peptide aggregation. Structurally, the PAS is lined with a cluster of aromatic residues, whose π-electron–rich rings form an entry path to the CAS [56]. These features facilitate interactions with Aβ peptides, and thus, blocking the PAS can effectively reduce amyloid aggregation while maintaining cholinergic enhancement. Examples include hybrid molecules linking donepezil or tacrine analogues to moieties such as coumarin or benzofuran [57]. In addition, in silico, in vitro [58,59,60] and in vivo [55] studies have proposed a range of alternative hybrid scaffolds further underscoring the structural diversity and future potential of this dual-targeting approach.

A good example of MLD is the dual-target inhibitors, where AChE is combined with other disease-relevant targets [11] such as monoamine oxidase B (MAO-B) [61], β-secretase (BACE-1) [62], glycogen synthase kinase-3β (GSK-3β) [63], or phosphodiesterases (PDEs) [64,65]. These targets can be classified as pathology-linked enzymatic targets in AD, each associated with distinct yet converging mechanisms beyond cholinergic dysfunction. Such designs pose fundamental structural challenges because the active sites of these enzymes are structurally disparate, and pharmacophores optimized for AChE are generally incompatible with the substrate-binding pockets of MAO-B, BACE-1, or GSK-3β. Consequently, dual-target ligands are predominantly engineered as either linked hybrids, wherein known inhibitors are joined via flexible or rigid linkers, or as multifunctional scaffolds incorporating chemical moieties capable of modest interactions across distinct targets. For instance, donepezil–propargylamine hybrids combine the AChE-binding functionality of donepezil with the MAO-B inhibitory propargylamine group; such compounds have demonstrated dual enzymatic activity in vitro [66,67]. However, dual-target BACE-1 or GSK-3β hybrids are comparatively limited, largely due to increased molecular weight, reduced blood–brain barrier permeability, and synthetic complexity. As a result, the majority of cross-target MTDL programs remain preclinical, with only limited advancement toward clinical evaluation, and some have been discontinued owing to unfavorable pharmacokinetic or physicochemical properties.

A notable extension of the MLDs strategy is the integration of metal-chelating functionalities into AChE inhibitor frameworks [68,69,70]. This approach responds to the increasing awareness of metal ion dysregulation in neurodegenerative diseases, where metals such as Cu^2+^, Fe^2+^, and Zn^2+^ accelerate Aβ aggregation and oxidative stress [71]. By embedding chelating moieties such as hydroxypyridinones and cyclen derivatives, or other metal-binding units into multifunctional ligands, researchers aim to develop compounds capable of both AChE inhibition and metal detoxification [72,73]. While much of the research has focused on extracellular Aβ, it is important to consider the intracellular protein tau, which undergoes hyperphosphorylation in disease states. In this process, phosphate groups are added to serine, threonine, and occasionally tyrosine residues, increasing the negative charge of tau and creating strong electrostatic complementarity for polyvalent cations such as Cu^2+^, Fe^2+^, Zn^2+^, and Al^3+^ [74]. These metals can coordinate with negatively charged phosphate groups and carboxylate side chains, promoting tau condensation and nucleation of filamentous aggregates [75]. The intracellular localization of tau, in compartments where metal ions are more concentrated, further facilitates these interactions. In contrast, Aβ aggregation occurs extracellularly, where metal ion concentrations are lower and direct coordination is less favoured, making metal-mediated tau aggregation potentially more efficient [76]. This mechanistic insight suggests that metal-chelating multifunctional ligands could provide dual benefits: reducing extracellular Aβ aggregation while also mitigating intracellular tau pathology. By targeting both processes, such compounds could address multiple pathological pathways, emphasizing the importance of considering tau-metal interactions in the design and evaluation of metal-targeted therapeutics in neurodegenerative disease.

Another significant strategy is Hybrid Ligand Design, which involves the structural fusion of two or more pharmacophores—natural, synthetic, or both—into a single molecule to enhance multitarget activity and therapeutic potential [77]. A current emphasis in this approach is the use of bioactive natural scaffolds, such as curcumin, flavonoids, and alkaloids, due to their intrinsic antioxidant, anti-amyloid, and neuroprotective properties [78,79]. These natural structures serve as versatile templates that can be chemically modified and combined with synthetic pharmacophores to yield multifunctional AChE inhibitors [80,81,82,83]. While natural scaffolds are a valuable component, the innovation of hybrid ligand design is not confined to them; entirely synthetic pharmacophores can also be strategically combined to achieve comparable or even superior multitarget functionality. Techniques such as molecular hybridization, scaffold hopping, semi-synthetic modification, and rational linker design are commonly used to fine-tune these compounds for improved pharmacokinetics, binding selectivity, and brain permeability. Notable examples include curcumin [84,85] and galantamine [86,87] based hybrids [88], which integrate potent cholinergic activity with additional neuroprotective mechanisms.

To overcome challenges related to BBB permeability and systemic toxicity, prodrug design has emerged as a valuable strategy [89,90,91,92]. This involves synthesizing inactive precursors equipped with brain-targeting promoieties that undergo enzymatic cleavage within the central nervous system to release the active inhibitor. Techniques such as lipophilic masking of polar groups have been employed to enhance CNS delivery [93,94].

Photopharmacology represents an approach that introduces light-responsive molecular switches into AChE inhibitors, enabling precise spatial and temporal control of enzyme inhibition [95]. Photo-switchable groups like azobenzene allow reversible modulation of inhibitor activity upon exposure to specific wavelengths of light. This innovative method has been demonstrated in azobenzene and dithienylethene (DTE) based hybrids and photoresponsive donepezil analogues, offering new possibilities for targeted therapy with minimized off-target effects [22,96,97].

Furthermore, advances in computational methods and artificial intelligence have revolutionized pharmacophore modelling and screening. Machine learning algorithms can analyse vast chemical libraries to predict novel AChE inhibitors with optimal binding profiles and favourable pharmacokinetics [98,99,100]. These tools accelerate the identification of candidates that combine efficacy with safety and brain penetrance, streamlining the drug development pipeline [101]. A summary of innovative approaches to AChE ligand design is presented in Table 3, which classifies the main strategies along with representative examples and key advantages.

### Comparison Between Dual-Site Inhibitors and Anti-Aggregation Strategies

Dual-site inhibitors and anti-aggregation strategies share the overarching objective of mitigating Alzheimer’s disease pathology by reducing the neurotoxic burden of Aβ and preserving neuronal function. Both approaches target Aβ aggregation, aiming to prevent synaptic dysfunction, attenuate neuroinflammatory responses, and slow cognitive decline, thereby representing disease-modifying interventions rather than purely symptomatic therapies [102].

Despite this shared therapeutic aim, the two strategies diverge in molecular targets and mechanisms of action as summarized in Table 4. Dual-site inhibitors engage both the CAS and PAS of AChE, thereby enhancing enzymatic inhibition and concurrently preventing AChE-mediated facilitation of Aβ aggregation. These agents provide dual therapeutic benefits by improving cholinergic neurotransmission while selectively interfering with enzyme-mediated amyloid aggregation [55], although their activity is largely restricted to AChE-dependent pathways and may be limited.

In contrast, anti-aggregation strategies act directly on Aβ monomers, oligomers, or fibrils to disrupt self-assembly and destabilize β-sheet structures. These interventions do not directly enhance cholinergic function but offer a broader spectrum of anti-amyloid activity [103]. Clinical challenges include limited BBB penetration, immune-related adverse effects, and modest symptomatic benefit.

Both strategies converge on attenuating amyloid-induced neurotoxicity; however, dual-site inhibitors integrate cholinergic enhancement with selective anti-aggregation, whereas anti-aggregation agents target Aβ assemblies directly, offering a broader but clinically more complex therapeutic profile.

**Table 4:** Overview of dual-site inhibitors and anti-aggregation strategies.

Feature	Dual-Site Inhibitors	Anti-Aggregation Strategies	References
Biological target	AChE (CAS + PAS)	Aβ peptides (monomers, oligomers, fibrils)	[55,104]
Mechanism of action	Inhibits ACh hydrolysis, enhancing cholinergic transmission Binds PAS to block AChE-facilitated Aβ aggregation	Prevents β-sheet formation; destabilizes oligomers/fibrils Promotes clearance via immune-mediated mechanisms	[103,104]
Representative classes of compounds	Acridine derivatives Aromatic amines/Benzylamines Carbamates Coumarins Indanones Piperidines/Piperazines Tacrine hybrids/Dimeric heteroaryls	Aldehydes Anthracycline Monoclonal antibody Peptide Polyphenol Single domain antibodies Sterols Tetracyclines	[103,105,106,107,108,109]
Therapeutic effect	Symptomatic improvement through cholinergic enhancement; disease-modifying effect by reducing Aβ aggregation	Primarily disease-modifying; reduces plaque load and toxic oligomers; limited immediate symptomatic benefit	[55,103,110]
Scope of activity	Targeting AChE-mediated pathways and partial Aβ aggregation	Targeting Aβ aggregation independently of enzymatic activity; some approaches also affect tau pathology	[55,102,103]
Limitations	Limited efficacy against Aβ aggregation independent of AChE activity; off-target cholinergic effects, including bradycardia and gastrointestinal disturbances Variable BBB penetration Narrow therapeutic index	Restricted BBB penetration, particularly for monoclonal antibodies Risk of immune-mediated adverse events, including amyloid-related imaging abnormalities (ARIA) Modest symptomatic benefit despite reduction in amyloid burden Limited selectivity of small-molecule agents for toxic Aβ species Efficacy dependent on disease stage and patient-specific factors	[55,102,103]

## 5. Experimental Techniques Driving Pharmacophore Validation

The validation of computational pharmacophore models has been enhanced over the years by experimental techniques that provide structural, kinetic, thermodynamic, and functional data. These methodologies helped predict binding interactions and guide model refinement but also enable the discovery of novel binding sites, elucidate conformational dynamics, characterize binding kinetics and thermodynamics, and provide functional validation in biologically relevant systems. Collectively, these approaches strengthen the translational value of pharmacophore-based inhibitor design.

X-ray crystallography remains the gold standard for elucidating high-resolution structures, offering atomic-resolution insights into AChE-inhibitor complexes. It enables the identification of key residues involved in ligand binding, the characterization of hydrogen bond donors/acceptors, hydrophobic interactions, and the observation of conformational adaptations upon ligand engagement. These data are instrumental in validating and refining spatial pharmacophore features, including the precise positioning of interaction points [111,112,113].

Cryo-electron microscopy (cryo-EM) is a tool used for studying large, flexible, or transient macromolecular assemblies that are often recalcitrant to crystallization. Cryo-EM provides structural snapshots across various conformational states, thus offering information on the dynamic nature of AChE and its allosteric regulation [114,115].

Surface plasmon resonance (SPR) and isothermal titration calorimetry (ITC) serve complementary roles in the characterization of ligand binding. SPR provides real-time data on the kinetics of association and dissociation, allowing the derivation of rate constants and equilibrium dissociation constants, which are vital for optimizing ligand affinity and residence time [116,117]. In parallel, ITC affords a thermodynamic profile of the binding interaction by quantifying changes in enthalpy (ΔH), entropy (ΔS), and Gibbs free energy (ΔG) [118,119], thereby elucidating the physicochemical driving forces behind ligand-receptor recognition [120].

Microfluidic technologies and biosensor-based platforms facilitate the high-throughput screening and real-time monitoring of AChE enzymatic activity and inhibitor efficacy. These miniaturized systems allow for multiplexed compound testing with low reagent consumption and high sensitivity, supporting early-phase pharmacophore validation and lead prioritization [121,122].

Cell-based assays, particularly those using human induced pluripotent stem cell (iPSC)-derived neuronal models, provide a physiologically relevant in vitro system and allow functional validation of compounds in a biologically relevant CNS-like environment [123]. These models capture essential features of the human CNS microenvironment and are critical for evaluating compound efficacy, neurotoxicity, blood–brain barrier permeability, and potential off-target effects, which are often overlooked in cell-free assays [124].

Integration of these experimental platforms forms an integrated framework that complements and informs computational pharmacophore modelling for reliable pharmacophore validation and optimization.

## 6. Challenges and Future Directions

Despite substantial progress in the development of advanced pharmacophore-based AChE inhibitors, several critical challenges continue to hinder their successful translation into clinically effective therapies for neurodegenerative disorders. One of the most persistent issues lies in achieving an optimal balance between multifunctionality, such as dual or multi-target inhibition, and favourable drug-like properties. Designing molecules that effectively engage multiple pathological targets while maintaining appropriate physicochemical characteristics (e.g., molecular weight, lipophilicity, and polarity) is inherently complex. These attributes influence CNS bioavailability, especially with regard to crossing the highly selective BBB.

The BBB remains a major pharmacological obstacle. While certain pharmacophores exhibit potent in vitro activity, many fail to achieve therapeutically relevant concentrations in the CNS due to poor permeability or active efflux mechanisms [125]. Therefore, strategies that improve CNS bioavailability while maintaining target specificity and favourable pharmacokinetic profiles are critically important.

Another critical challenge involves the mitigation of off-target interactions and the minimization of metabolic liabilities [126]. Many multi-functional compounds tend to exhibit assorted binding, which can lead to unwanted side effects, toxicity, or rapid metabolic degradation [127]. Enhancing specificity while retaining therapeutic breadth demands advanced strategies in rational design and predictive ADME (Absorption, Distribution, Metabolism, and Excretion) modelling.

Looking forward, several promising avenues are emerging to address these multifaceted challenges. Artificial intelligence (AI) driven de novo drug design is rapidly transforming the pharmacophore discovery process [128]. Machine learning algorithms are now capable of exploring vast chemical spaces, predicting binding affinities, and generating novel scaffolds with optimized pharmacokinetic and pharmacodynamic properties [129]. These tools enable more efficient identification of lead compounds with desired characteristics.

Simultaneously, advanced drug delivery systems—such as nanoparticle-based carriers [130,131], intranasal formulations [132], and prodrugs—are being explored to improve CNS delivery and reduce systemic exposure. These technologies offer the potential to overcome traditional pharmacokinetic limitations and enhance therapeutic efficacy.

Ultimately, the successful development of next-generation AChE inhibitors will require sustained interdisciplinary collaboration. Integrating expertise from structural biology, medicinal chemistry, computational modelling, pharmacology, and clinical sciences will be essential. Only through such synergistic efforts can we hope to translate innovative pharmacophore concepts into safe, effective, and individualized therapies for complex CNS disorders.

## 7. Conclusions

The design of next-generation acetylcholinesterase inhibitors has shifted decisively toward multifunctional, disease-modifying strategies that go beyond symptomatic cholinergic enhancement. Of particular importance is the growing recognition of AChE’s role in β-amyloid aggregation via its PAS, which repositions the enzyme as a critical modulator in the amyloid cascade. Novel pharmacophores—especially dual-site inhibitors and MTDLs—are being designed to both inhibit enzymatic activity and interfere with Aβ fibrillization, often integrating antioxidant or metal-chelating functionalities. Furthermore, AI-driven screening, structure-based design, and validation in human iPSC-derived neuronal models ensure that ligand development increasingly reflects physiologically relevant conditions. This convergence of molecular innovation with disease-contextual validation represents a promising path toward clinically viable AChE-targeted therapies capable of modifying core features of Alzheimer’s pathology.

## Figures and Tables

**Table 1:** Structural domains of human AChE.

Domain	Location	Key Amino Acids	Functions	References
Catalytic Active Site (CAS)	Bottom of the active-site gorge	S203, H447, E334, W86, Y337	Catalyzes the rapid hydrolysis of AChE via the catalytic triad Trp86 stabilizes the quaternary ammonium group Tyr337 functions as a conformational gate	[30,31]
Peripheral Anionic Site (PAS)	Entrance of the active-site gorge	Y72, B74, Y124, W286, Y341	Facilitates initial substrate recognition and guidance into the active-site gorge W286 and Y341 mediate π-π interactions with aromatic ligands B74 and Y124 contribute to electrostatic and hydrogen-bond interactions that facilitate substrate pre-orientation	[14,32,33]
Oxyanion Hole (OH)	Near CAS within the active-site gorge	G121, G122, A204	Stabilizes the high-energy tetrahedral intermediate during catalysis by forming hydrogen bonds with the negatively charged oxygen atom	[34]
Acyl Pocket	Within the CAS region	F295, F297	Provides steric specificity for the acyl moiety of the substrate, contributing to the enzyme’s substrate selectivity	[31]

**Table 2:** Summary of clinically approved acetylcholinesterase inhibitors and their limitation.

Inhibitor	Mechanism	Clinical Benefit	Limitations	References
Donepezil	Reversible AChE inhibitor	Temporary cognitive improvement	Peripheral side effects, limited disease modification; gastrointestinal adverse effects include nausea, diarrhea, and vomiting	[38,41,43,47]
Galantamine	Reversible AChE inhibitor and allosteric modulator of nAChRs	Temporary cognitive improvement	Gastrointestinal adverse effects include loss of appetite, nausea, vomiting, diarrhea, and weight loss; clinical use may also be influenced by variability in central nervous system bioavailability related to formulation and patient-specific factors	[39,40,43,48]
Rivastigmine	Pseudo-irreversible AChE and BuChE inhibitor	Temporary cognitive improvement	Gastrointestinal adverse effects include nausea, vomiting, weight loss, and diarrhea, compounded by challenges related to dosing complexity	[38,42,43,49]

**Table 3:** Strategies for designing novel acetylcholinesterase ligands.

Strategy	Description	Representative Examples	Key Advantages	References
Conventional	Direct enzyme blockade enhancing cholinergic neurotransmission via competitive or non-competitive inhibition.	Donepezil, Rivastigmine, Galantamine	Well-established efficacy Direct and specific cholinergic enhancement with proven clinical utility	[38,39,41,42,43]
Dual site	Ligands targeting both CAS and PAS of AChE for enhanced inhibition and anti-amyloid activity.	Donepezil–benzofuran hybrids, Tacrine–coumarin hybrids, Bis-(7)-tacrine	Improved inhibitory efficacy and binding affinity Dual modulation of enzymatic activity and amyloidogenic pathways	[51,55,61,86]
Multi-target-directed ligands	Single molecules modulating AChE and other AD-related targets	Donepezil–MAO-B inhibitors, Tacrine–BACE-1 hybrids, Galantamine–GSK-3β conjugates, Coumarin–PDE inhibitors	Multifunctional therapeutic potential addressing multiple pathogenic mechanisms including cholinergic dysfunction, oxidative stress, and amyloidogenesis	[62,63,64,65,70,87,88]
Chelators	AChE inhibitors incorporating metal-chelating groups to mitigate metal-induced Aβ aggregation and oxidative stress.	Hydroxypyridinone derivatives, Cyclen-based AChE ligands, Tacrine–metal chelator conjugates	Dual activity combining enzymatic inhibition with metal ion detoxification, allowing mitigation of metal-induced neurotoxicity	[68,69,70,71,72,73]
Hybrid ligand design	Fusion of natural and/or synthetic pharmacophores into single molecules to enhance multitarget efficacy and pharmacokinetics.	Curcumin–tacrine hybrids, Galantamine–flavonoid conjugates, Tacrine–resveratrol hybrids	Enhanced multitarget efficacy Improved blood–brain barrier penetration, selectivity, and neuroprotective potential	[77,78,79,80,81,82,83,84,85,86,87,88]
Prodrug	Enzymatically activated inactive precursors designed to improve CNS delivery and reduce systemic toxicity.	Galantamine carbamates, Tacrine–prodrugs with lipophilic masking groups	Improved blood–brain barrier permeability Minimized systemic adverse effects through site-specific activation	[89,90,91,92,93,94]
Photopharmacology	Light-activated inhibitors with reversible control of AChE inhibition via photo-switchable molecular groups.	Azobenzene-based donepezil analogs, Dithienylethene (DTE)–tacrine hybrids, Photoresponsive tacrine derivatives	Precise spatiotemporal control of inhibition; potential reduction in off-target effects and enhanced therapeutic selectivity	[22,95,96,97]

## Abbreviations

The following abbreviations are used in this manuscript:

ACh	Acetylcholine
AChE	Acetylcholinesterase
AD	Alzheimer’s Disease
ADME	Absorption, Distribution, Metabolism, and Excretion
AI	Artificial Intelligence
BACE-1	Beta-site Amyloid Precursor Protein Cleaving Enzyme 1
BBB	Blood–Brain Barrier
CAS	Catalytic Active Site
CNS	Central Nervous System
DTE	Dithienylethene
EMA	European Medicines Agency
FDA	U.S. Food and Drug Administration
GSK-3β	Glycogen Synthase Kinase 3 Beta
iPSC	Induced Pluripotent Stem Cell
ITC	Isothermal Titration Calorimetry
MAO-B	Monoamine Oxidase B
MTDLs	Multi-Target-Directed Ligands
OH	Oxyanion Hole
PAS	Peripheral Anionic Site
PDEs	Phosphodiesterases
SPR	Surface Plasmon Resonance
